# Validation of the XDP–MDSP rating scale for the evaluation of patients with X-linked dystonia-parkinsonism

**DOI:** 10.1038/s41531-017-0026-0

**Published:** 2017-07-25

**Authors:** Paul Matthew D. Pasco, Roland Dominic G. Jamora, Raymond L. Rosales, Cid Czarina E. Diesta, Arlene R. Ng, Rosalia A. Teleg, Criscely L. Go, Lillian Lee, Hubert H. Fernandez

**Affiliations:** 1 Department of Neurosciences, College of Medicine-Philippine General Hospital, University of the Philippines Manila, Manila, Philippines; 2 Child Neuroscience Center, Philippine Children’s Medical Center, Quezon City, Philippines; 3 Movement Disorders Service and Section of Neurology, Institute for Neurosciences, St. Luke’s Medical Center, Quezon City and Global City, Philippines; 4 Department of Neurology and Psychiatry, University of Santo Tomas Hospital, Manila, Philippines; 5 Center for Neurodiagnostic and Therapeutic Services, Metropolitan Medical Center, Manila, Philippines; 6 Department of Neurosciences, Movement Disorders Clinic, Makati Medical Center, Makati City, Philippines; 7 Section of Neurology, Department of Internal Medicine, National Kidney and Transplant Institute, Quezon City, Philippines; 8 Departments of Neurology and Behavioral Medicine, Dr. Jose R. Reyes Memorial Medical Center, Manila, Philippines; 9 Center for Neurological Restoration, Cleveland Clinic, Cleveland, OH USA; 10 Cleveland Clinic Lerner College of Medicine, Cleveland, OH USA

## Abstract

X-linked dystonia-parkinsonism(XDP) is a neurodegenerative disorder endemic to the Philippines. A rating scale was developed by the authors under the guidance of the Movement Disorder Society of the Philippines (MDSP) to assess XDP severity and progression, functional impact, and response to treatment in future clinical trials. Our main objective was to validate our new scale, the XDP–MDSP scale. The initial validation process included pragmatic testing to XDP patients followed by a modified Delphi procedure with an international advisory panel of dystonia, parkinsonism and scale development experts. Pearson correlation was used to assess construct validity of our new scale versus the assess construct validity of our new scale versus standard dystonia, parkinsonism, non-motor and functional scales; and also to assess divergent validity against behavioral and cognitive scales. The 37-item XDP–MDSP scale has five parts: I-dystonia, II-parkinsonism, III-non-motor features, IV-ADL, and V-global impression. After initial validation, the scale was administered to 204 XDP patients. Inter-domain correlation for the first four parts was acceptable. The correlation between these domains and the global rating was slightly lower. Correlations between Parts I, II, III, and IV versus standard dystonia, parkinsonism, non-motor and functional scales were acceptable with values ranging from 0.323 to 0.428. For divergent validity, a significant correlation was seen with behavioral scales. No significant correlation was noted with the cognitive scale. The proposed XDP–MDSP scale is internally valid but the global rating subscale may need to be modified or eliminated. While there is convergent validity, divergent validation was successful only on cognitive and not behavioral scales. The frequent co-occurrence of anxiety and depression, and its effect on the motor and functional state, may explain this finding.

## Disease rating scale: Adressing a Philippine problem

Researchers validate a tool to assess patients with X-linked dystonia parkinsonism (XDP), a movement disorder that affects men of Filipino descent. Roland Dominic Jamora and Paul Matthew Pasco, at the University of the Philippines Manila, and colleagues collaborated with the Movement Disorder Society of the Philippines (MDSP) to develop a new comprehensive rating scale that is specific for this condition. The 37-item test was administered to over 200 patients and the results correlated with those obtained in commonly used scales for the assesment of dystonia, parkinsonism, motor and non-motor symptoms. The authors conclude that the XDP-MDSP rating scale will allow clinicians and researchers to evaluate the severity of XDP as well as determine patients’ response to treatment. Further refining the scale may be required to discern the effect of patients’ anxiety and depression on their functional state.

## Introduction

X-linked dystonia-parkinsonism (XDP, DYT3, “Lubag”, OMIM #314250) is an adult-onset, progressive, neurodegenerative movement disorder first described in Filipino males from Panay Islands in 1975, and so far found only in Filipinos.^1^ The nationwide prevalence is 0.31/100,000, but is 23.66/100,000 in Capiz and 7.72/100,000 in Aklan. The mean age at onset of illness is 39.67 years and the mean age at death is 55.59 years. Only 6% of survivors are still able to work, 69% are ambulant but not working due to the profound disability caused by the movement disorders, and 23% are wheelchair-bound or bed-bound.^2^

XDP typically manifests initially with focal dystonia (93%), and initial parkinsonian traits are observed in only 5.7%. The condition generalizes within 5 years of onset in 84% of cases, regardless of the initial site of involvement. As the illness reaches from the 7th to 10th year, the dystonic movements become less severe, with apparent stiffening of the limbs and straightening of the trunk. By the 15th year of illness, the predominant picture is one of parkinsonism manifesting as bradykinesia, masked facies, mumbling speech with drooling, and tremors.^2, 3^

There are currently no existing scales specific for XDP, a major impediment in having objective means of classifying patients and the extent of their disease severity, tracking disease progression and response to treatment. Hence the authors, in cooperation with the Movement Disorder Society of the Philippines (MDSP) developed this scale for clinical and research use.

A proposed scale for use in patients with XDP is presented, with the intention of validating it for clinical use. A validated scale will be useful to clinicians who manage patients with XDP and clinical researchers that test effects of various interventions for uniformity in their assessments.

## Results

A total of 204 patients with a clinical diagnosis of XDP were recruited to the study. Cronbach’s alpha for the entire five-part scale was acceptable at 0.805. Inter-domain correlation for the first four parts of the scale measuring four different domains (dystonia, parkinsonism, non-motor features and activities of daily living) was acceptable and significant with ranges from 0.434 to 0.671; the correlation between these domains and the last (global rating) was slightly lower but still significant at 0.319 to 0.447 (Table 1).

**Table 1:** Inter-domain correlation of the different domains of the XDP–MDSP scale (I: dystonia; II: parkinsonism; III: non-motor features; IV: activities of daily living; V: overall assessment)

		I	II	III	IV	V	Whole Scale
I	Pearson Correlation	1	.578^**^	.570^**^	.671^**^	.447^**^	.853^**^
	Sig. (2-tailed)		.000	.000	.000	.000	.000
II	Pearson Correlation	.578^**^	1	.434^**^	.587^**^	.319^**^	.825^**^
	Sig. (2-tailed)	.000		.000	.000	.000	.000
III	Pearson Correlation	.570^**^	.434^**^	1	.615^**^	.328^**^	.746^**^
	Sig. (2-tailed)	.000	.000		.000	.000	.000
IV	Pearson Correlation	.671^**^	.587^**^	.615^**^	1	.425^**^	.861^**^
	Sig. (2-tailed)	.000	.000	.000		.000	.000
V	Pearson Correlation	.447^**^	.319^**^	.328^**^	.425^**^	1	.514^**^
	Sig. (2-tailed)	.000	.000	.000	.000		.000
WHOLE SCALE	Pearson Correlation	.853^**^	.825^**^	.746^**^	.861^**^	.514^**^	1
	Sig. (2-tailed)	.000	.000	.000	.000	.000	

**Correlation is significant at the 0.01 level (2-tailed)

For pragmatic validity, the average time spent completing all five parts of the scale was 40 min. Inter-rater validation was no longer pursued when it was determined that too only 7 of the 37 items on the scale could be evaluated using the video recordings.

For convergent validity, correlation was significant and acceptable with values ranging from 0.323 to 0.428 (for XDP–MDSP Scale Part I and BFMDRS, Part II and UPDRS motor, Part III and NMSQuest and Part IV and SCOPA-ADL) (see Table 2). When testing for divergent validity, there was a trend toward negative correlation between the overall XDP–MDSP scale score, and the MMSE and a significant positive correlation with the HADS-P and HAM-D. If only Parts I, II, and IV of the XDP scale (which do not contain behavioral or cognitive items) are examined, there is now a trend of no correlation with the MMSE and HAM-D but a significant correlation with the HADS-P remains.

**Table 2:** Determination of convergent validity via correlation of the different domains of the XDP–MDSP scale with various external scales (I with BFMDRS, II with UPDRS motor, III with NMSQuest and IV with SCOPA) and correlation with HADS-P, HAMD and MMSE

		BFMDRS	UPDRS	NMS	SCOPA	MMSE	HADS-P	HAM-D
I	Pearson Correlation	.428^**^	.266^**^	.189^*^	.349^**^	−.118	.306^**^	.176^*^
	Sig. (2-tailed)	.000	.000	.012	.000	.120	.000	.021
	N	184	181	176	183	176	183	171
II	Pearson Correlation	.183^*^	.323^**^	.135	.312^**^	−.083	.156^*^	.114
	Sig. (2-tailed)	.013	.000	.073	.000	.271	.035	.136
	N	184	181	176	183	176	183	171
III	Pearson Correlation	.358^**^	.238^**^	.335^**^	.340^**^	−.035	.276^**^	.362^**^
	Sig. (2-tailed)	.000	.001	.000	.000	.640	.000	.000
	N	184	181	176	183	176	183	171
IV	Pearson Correlation	.326^**^	.301^**^	.185^*^	.396^**^	−.137	.231^**^	.142
	Sig. (2-tailed)	.000	.000	.014	.000	.069	.002	.064
	N	184	181	176	183	176	183	171
XDP	Pearson Correlation	.381^**^	.345^**^	.246^**^	.413^**^	−.137	.289^**^	.227^**^
	Sig. (2-tailed)	.000	.000	.001	.000	.069	.000	.003
	N	184	181	176	183	176	183	171

*Correlation is significant at the 0.05 level (2-tailed)

**Correlation is significant at the 0.01 level (2-tailed)

## Discussion

This is to our knowledge, the first comprehensive assessment scale on XDP ever reported and validated that comprised a section for dystonia, parkinsonism, non-motor features, activities of daily living and global assessment. Although the XDP–MDSP scale takes on average 40 min to administer, it eliminates the need to administer separate dystonia, parkinsonism, non-motor and functional scales, which cumulatively can take longer than 40 min. Also, since not all patients may have all features, the entire scale can often take less time to complete.

While the Cronbach’s alpha for the entire five-part scale was acceptable at 0.805, and the inter-domain correlation for the first four parts of the scale measuring four different domains (dystonia, parkinsonism, non-motor features and activities of daily living) was acceptable and significant; the correlation between these domains and Part V—the global rating was slightly lower but still significant. This implies that the global impression subscale of the XDP–MDSP scale may need to be modified. The subscale was heavily based on the Clinical Global Impression Scale (CGIS), a one-item, 7-point scale, ranging from normal (no disease symptoms) to extremely ill (among the worst disease severity encountered). The CGIS may therefore not be detailed enough or specific enough when evaluating for disease severity in this unique population, which may account for the lower correlation of this domain with the rest of the scale.

For convergent validity, the correlation between the Parts I–IV of the XDP–MDSP scale and their corresponding gold standard counterparts was significant and acceptable. However, when testing for divergent validity, the desired negative/no correlation was barely met with the MMSE (a trend was seen) but not with the HADS-P and HAM-D (where significance was noted).

Nonetheless, if only items of the XDP–MDSP scale which do not contain behavioral or cognitive items were examined, there is no longer a correlation with the MMSE and HAM-D, but a significant correlation with the HADS-P remains. This was probably because XDP patients have a concomitant anxiety and depression. Studies have shown a prevalence of anxiety symptoms at 16.7% and depressive symptoms between 54.8–92.9% among XDP patients.^4^ This again serves as a warning that future studies that evaluate motor improvement in this population should look carefully at the intervention’s effect on behavior as they can be a significant confounder.

The potential weaknesses in this validation study are the lack of controls and our inability to take proactive measures to minimize skewness, floor, and ceiling effects. Since XDP is a rather uncommon disorder, endemic to only certain areas in the Philippine archipelago, we had to simply enroll as many patients as we could without much regard to their disease severity. Nonetheless, to minimize the effects of skewness, we enrolled more patients than what is thought to be typical for the scale’s length. Our distribution and range of scores are also described in our Result section. Moreover, most other recently validated movement disorders scales have also not included controls or emphasized skewness, floor and ceiling effects.

## Conclusion

The proposed XDP–MDSP scale is internally valid, although the last domain (global rating) should perhaps be modified slightly due to lower correlation with the other domains. Since Pearson correlation is acceptable, there is also convergent validity. On the other hand, the significant correlation between the proposed scale and the HADS-P and HAM-D may be because many patients also have concomitant anxiety and depression. Correlation with only those parts of the scale that do not contain behavioral or cognitive items shows partially successful divergent validation.

The acceptable internal validity of the scale and convergent validity make this proposed XDP–MDSP scale valid and acceptable to assess the severity of the disease as well as the patient’s response to treatment. Subsequent studies, such as clinical trials in this population can make use of the scale in order to assess the effectiveness of treatments as well as the natural history of the disease. Patient satisfaction with the scale can also be assessed more thoroughly by means of a questionnaire or survey aside from the pilot testing that was done. This is planned for a future validation phase, which will involve more patients when using the scale in clinical trials or progression studies.

## Methods

The authors developed the scale based on their collective clinical experience, with the aim of capturing the various phases of the illness, its associated non-motor features, along with a global impression subscale that could be useful in tracking progression and response to treatment.

The investigators used the most commonly accepted gold standard scales in the assessment of various aspects of XDP. In assessing dystonia, the Burke–Fahn–Marsden dystonia rating scale (BFMDRS) was used.^5^ For PD, the Unified Parkinson Disease Rating Scale (UPDRS) motor was used.^6^ For the non-motor symptoms, the Non-Motor Symptoms Questionnaire (NMSQuest) was used.^7^ The Short Parkinson’s Evaluation Scale/Scales for Outcomes in Parkinson’s Disease (SPES/SCOPA) was utilized to look into the motor impairments, activities of daily living, and motor complications.^8^

Construct validity was assessed by correlating the different parts of the scale with the following validated scales: BFMDRS, UPDRS Motor, NMSQuest and SCOPA-ADL subscale. The XDP–MDSP scale’s internal consistency was assessed by computing for the Cronbach’s alpha coefficient.

To ensure that the XDP–MDSP scale was not unduly influenced by the presence of confounders, such as depression, anxiety and cognitive impairment, divergent validity was assessed by correlating the XDP–MDSP scale with the Hospital Anxiety and Depression Scale-Pilipino (HADS-P), the Hamilton Depression Rating Scale (HAM-D) and the Mini-Mental State Examination.^9–11^

After the scale was completed, a prospective, cross-sectional validation study was done. The patients were recruited from the investigators’ clinics in Manila and from the XDP Clinic in Roxas City, Capiz. Patients with a clinical diagnosis of XDP based on the following were included: male sex, family history of dystonia and/or parkinsonism, inheritance pattern consistent with an X-linked recessive pattern and patients with whatever combination or severity of dystonia and/or parkinsonism. We excluded patients whose signs and symptoms could be explained by another diagnosis aside from XDP.

The initial version of the XDP–MDSP rating scale was first applied to ten XDP patients for initial feedback. The scale was shortened after it was found to be too long and exhaustive for both the clinicians and the patients. In addition, certain items found to be vague were clarified and refined.

Further content validation was then carried out using the modified Delphi technique. An international advisory panel was formed, composed of five movement disorders experts. Their comments on the scale were individually solicited. Further modifications to the scale were made based on their comments. The technique overcomes the disadvantages of conventional committee action (e.g., censored feedback) by allowing experts to provide relatively anonymous feedback. The revised version of the scale was again administered to a small group of patients to examine if they understood the individual items.

The final XDP–MDSP scale consisted of 37 items divided into five parts to capture all XDP-related symptoms: part 1—dystonia, part II—parkinsonism, part III A and B—non-motor symptoms, part IV—activities of daily living and part V—global impression. To achieve optimal analyses, the scale was administered to at least 200 XDP patients, based on an approximate sample size calculation of 37 scale items × 5 = 185 patients plus 15 additional patients to allow for possible dropouts and to minimize the influence of any unanticipated skewness, floor and ceiling effects, as much as possible. Proper informed consent was obtained prior to the start of the study. Only one rater evaluated each patient. The raters scored patients on each item according to his or her best judgment, as to which score best describes the patient. They asked the patients about any doubts or unclear items. The time spent to administer the scale to each patient was taken.

After the XDP–MDSP scale was completed for each patient, the BFMDRS, UPDRS motor, NMSQuest, SCOPA-ADL subscale, HADS-P, HAM-D, and MMSE were also administered. If the patient became fatigued or was unable to communicate due to his disease condition, the examiner allowed for time to rest or to ask the companion for information regarding the patient’s functioning.

The raters in the study were the authors, as well as other officers and members of the MDSP, who are all neurologists and experts in movement disorders. All raters underwent training and orientation in the administration of the XDP–MDSP scale and all other scales to be used, in order to clarify use and description of all instruments and standardize ratings.

The validity of the scale was assessed using the following: for pragmatic validity, the average time for completing the scale by both clinician and caregiver or patient was taken. For inter-rater validity, at least two raters scored the patients based on a video recording made using a standardized protocol. For construct (or convergent) validity, the correlation between the different parts of the XDP–MDSP scale and the BFMDRS, UPDRS Motor, NMSQuest, SCOPA-ADL was calculated. For divergent validity, the correlation between the XDP–MDSP scale and HADS-P, HAM-D and MMSE was calculated. The convergent and divergent validity were tested using Pearson correlation.

Some parts were clinician-administered (Parts I, II, IIIA, and V) and others were answered independently by the patient and/or caregiver (Parts IIIB and IV). The ratings were in whole integers. If the score lies between two items, the rater was advised to use the higher number.

The scores for all scales were entered into an Excel file, with appropriate quality control and data processing measures performed. For internal consistency, Cronbach’s alpha coefficient was calculated. The methods were performed in accordance to relevant regulations and guidelines. This study was approved by the Institutional Review Board of the Philippine Children’s Medical Center.

### Data availability

The data that support the findings of this study are available from the corresponding author upon reasonable request.

## Electronic supplementary material

XDP-MDSP RATING SCALE
