# Supplementary material for: Validation of the XDP–MDSP rating scale for the evaluation of patients with X-linked dystonia-parkinsonism
Source: NPJ Parkinsons Dis. 2017 Jul 25;3:24. doi: 10.1038/s41531-017-0026-0 (PMC5526880; doi:10.1038/s41531-017-0026-0)
Supplement: Supplementary file 1 — XDP-MDSP RATING SCALE [file 41531_2017_26_MOESM1_ESM.doc]

**XDP-MDSP RATING SCALE**

**Instructions to the Rater:**

OVERVIEW: This scale is designed to rate the severity of dystonic, parkinsonian and non-motor symptoms in patients with X-linked Dystonia Parkinsonism (XDP; DYT-3; Lubag) and their effect on activities of daily living. The ratings depend on using all available information, which is generally provided through the patient’s and caregiver’s report and the clinical interview. The scale is comprised of 5 subscales: Part I (*Dystonia*); Part II (*Parkinsonism*); Part III A and B (*Non-Motor Symptoms*); Part IV (*Activities of Daily Living*); and Part V (*Global Impression*). Some parts are clinician-administered (Parts I, II, IIIA, and V) and other parts are answered independently by the patient and/or caregiver (Parts IIIB and IV). *Unless otherwise indicated/instructed, rate as is*. Do not factor for age, co-morbidities or drug effects. For Parts III and IV, the characteristics of each item during the prior week, up until and including the time of the interview, should be rated. For all questions that rely on historical information, scores should reflect the *average*, defined as *most usual* or *most common* occurrence of each item for the entire week, unless specified otherwise.

INFORMANTS: For the clinician-administered portions of the scale, historical information may need to be obtained by interviewing either: 1) the patient, 2) the caregiver or, 3) the patient and caregiver together (recommended, especially clarifying differences if indicated). Information from each of these interviews should then be combined to help determine the scoring of each item. In the instance of conflicting information, the clinician should rely on the information believed to be most valid. Consistent reporting can be ensured by having the same informant(s) present for each rating session. Mark at the top of the form the primary data source (i.e. patient, caregiver, or patient and caregiver in equal proportion).

SCORING: All ratings should be in whole integers. If the score lies between two items, please rate the higher number. Add the scores in each item for the total score for each subscale. The *over-all score* is the sum of the total scores in each subscale. The scale is then followed by a Global Impression scale (global severity and global improvement), adapted from the Clinical Global Impression Scale (Guy, 1976) to provide measures of overall functional impairment.

**Patient ID: Site:**

**Date: Examiner:**

**Time of last medication intake: ________ Duration of Illness: _____________**

**PART I: DYSTONIA**

1. EYES AND UPPER FACE

**Specific Instructions**: Ask the patient (or caregiver) how often, in the past week, s/he has noticed increased blinking, forceful eye closure, or forehead wrinkling. Then ask the patient to tightly open and close eyes five times

0 = ***Normal*** AND ***None***: Absent signs and symptoms

1 = ***Slight*** AND ***Seldom***: Increased blinking or slight forehead wrinkling/ lid retraction AND dystonia over eyes and/or upper face occurring less than 26% of the time during waking hours in the past week

2 = ***Mild*** OR ***Often***: Increased blinking with some eye closure but without squeezing/ mild

forehead wrinkling OR dystonia over eyes and/or upper face occurring 26-50% of the time during waking hours in the past week

3 = ***Moderate*** OR ***Frequent***: Eye closure with squeezing but still within 10 seconds able to open eyes within 10 seconds/ with pronounced forehead wrinkling OR dystonia over eyes and/or upper face occurring 51-75% of the time during waking hours in the past week

4 = ***Severe***: OR ***All the time***: Eyes closed most of the time/ eye closure with squeezing, unable to open eyes within 10 seconds/ persistent forehead wrinkling with pronounced forehead lines OR dystonia over eyes and/or upper face occurring more than 75% of the time during waking hours in the past week

2. JAW, TONGUE AND LOWER FACE

**Specific Instructions**: Ask the patient (or caregiver) how often, in the past week, has s/he noted involuntary grimacing of the lower face, jaw or tongue protrusion/retraction. Then ask the patient to open and close the jaw five times; ask the patient to protrude, retract and move tongue side to side

0 = ***Normal*** AND ***None***: Absent signs and symptoms

1 = ***Slight*** AND ***Seldom***: Slight grimacing of lower face, easily able to relax/ slight jaw protrusion or retraction/ slight tongue protrusion or retraction AND dystonia over jaw, tongue or lower face occurring less than 26% of the time during waking hours in the past week

2 = ***Mild*** OR ***Often***: Mild grimacing of the lower face with minimal distortion of the mouth/ mild jaw protrusion or retraction, with some difficulty relaxing/ mild tongue

protrusion or retraction or rolling OR dystonia over jaw, tongue or lower face occurring 26-50% of the time during waking hours in the past week

3 = ***Moderate*** OR ***Frequent***: Grimacing of lower face with moderate distortion of the mouth/ moderate jaw protrusion or retraction/ spontaneous tongue protrusion beyond the lips***/*** difficulty retracting tongue to normal position/ cheek pushed due to tongue rolling OR dystonia over jaw, tongue or lower face occurring 51-75% of the time during waking hours in the past week

4 = ***Severe*** OR ***All the time***: Persistent grimacing of the lower face/ unable to relax jaw/ jaw dislocated/ unable retract or protrude tongue / tongue fully protruded OR dystonia over jaw, tongue or lower face occurring more than 75% of the time during waking hours in the past week

3. LARYNX

**Specific Instructions**: Ask the patient (or caregiver), how often, over the past week, has s/he

noticed having hoarseness, choked voice or voice breaks when talking. Converse with the patient,

then ask to vocalize “eeeee” for 5 seconds

0 = ***Normal*** AND ***None***: Absent signs and symptoms

1 = ***Slight*** AND ***Seldom***: Barely detectable hoarseness/ choked voice or voice breaks AND occurring less than 26% of the time

2 = ***Mild*** OR ***Often***: Obvious hoarseness/ frequent voice breaks and choked voice OR occurring 26 – 50% of the time

3 = ***Moderate*** OR ***Frequent***: Marked hoarseness/ choked voice or continuous voice breaks OR occurring 51-75% of the time

1. ***= Severe*** OR ***All the time***: Unable to vocalize / presence of audible respiratory grunts or wheezing / presence of tridor OR occurring more than 75% of the time

4. NECK AND SHOULDER (elevation, anterior and posterior displacement)

**Specific Instructions:** Ask the patient (or caregiver) how often, over the past week, has s/he noted involuntary neck deviation or shoulder elevation. Then ask the patient to walk at least 5 meters and observe neck rotation, flexion, extension and shoulder elevation, anterior and posterior displacement

0 = ***Normal*** AND ***None***: Absent signs and symptoms

1 = ***Slight*** AND ***Seldom***: Slight deviation of the neck and shoulder that occurs only with maneuvers AND occurring dystonia over neck and/or shoulders occurring less than 26% of the time during waking hours in the past week

2 = ***Mild*** OR ***Often***: Mild deviation of the neck and shoulder even without maneuvers OR dystonia over neck and/or shoulders occurring 26-50% of the time during waking hours in the past week

3 = ***Moderate*** OR ***Frequent***: Significant deviation of the neck and shoulder but still able to relax OR dystonia over neck and/or shoulders occurring less than 51-75% of the time during waking hours in the past week

4 = ***Severe*** OR ***All the time***: Fixed deviation of the neck and shoulder with inability to relax OR dystonia over neck and/or shoulders occurring more than 75% of the time during waking hours in the past week

5. UPPER AND LOWER TRUNK

**Specific Instructions**: Ask the patient (or caregiver) how often, over the past week, has s/he noticed involuntary rotation, deviation, flexion or extension of the trunk. Then observe the patient while lying down, seated, standing, and walking for at least 5 meters. Observe for rotation, lateral deviation, flexion or extension.

0 = ***Normal*** AND ***None***: Absent signs and symptoms

1 = ***Slight*** AND ***Seldom***: Slight deviation of the trunk that occurs only when walking AND dystonia over the trunk occurring less than 26% of the time during waking hours in the past week

2 = ***Mild* OR *Often***: Mild deviation of the trunk seen while lying down, seated or standing OR dystonia over the trunk occurring 26-50% of the time during waking hours in the past week

3 = ***Moderate*** OR ***Frequent***: Significant deviation of the trunk but still able to ambulate without assistance OR dystonia over the trunk occurring less than 51-75% of the time during waking hours in the past week

4 = ***Severe*** OR ***All the time***: Persistent deviation of the trunk and able to stand and ambulate only with assistance/ unable to maintain sitting position OR dystonia over the trunk occurring more than 75% of the time during waking hours in the past week

6A. RIGHT UPPER EXTREMITY

**Specific Instructions:** Ask the patient (or caregiver) how often, over the past week, has s/he noticed involuntary posturing of the right upper extremity. Then observe the patient with hands on the lap and ask the patient to fully extend the arms. Then with arms outstretched in front, ask the patient to fully pronate and supinate for five times

0 = ***Normal*** AND ***None***: Absent signs and symptoms

1 = ***Slight*** AND ***Seldom***: Slight posturing and seen only with maneuvers AND dystonia of the extremity occurring less than 25% of the time during waking hours in the past week

2 = ***Mild*** OR ***Often***: Mild posturing and seen even without maneuvers OR dystonia of the extremity occurring 26-50% of the time during waking hours in the past week

3 = ***Moderate*** OR ***Frequent***: Significant posturing is present with difficulty performing maneuvers OR dystonia of the extremity occurring less than 51-75% of the time during waking hours in the past week

4 = ***Severe*** OR ***All the time***: Fixed posturing and unable to perform maneuvers OR dystonia of the extremity occurring more than 75% of the time during waking hours in the past week

6B. LEFT UPPER EXTREMITY

**Specific Instructions:** Ask the patient (or caregiver) how often, over the past week, has s/he noticed involuntary posturing of the left upper extremity. Then observe the patient with hands on the lap and ask the patient to fully extend the arms. Then with arms outstretched in front, ask the patient to fully pronate and supinate for five times

0 = ***Normal*** AND ***None***: Absent signs and symptoms

1 = ***Slight*** AND ***Seldom***: Slight posturing and seen only with maneuvers AND dystonia of the extremity occurring less than 26% of the time during waking hours in the past week

2 = ***Mild*** OR ***Often***: Mild posturing and seen even without maneuvers OR dystonia of the extremity occurring 26-50% of the time during waking hours in the past week

3 = ***Moderate*** OR ***Frequent***: Significant posturing is present with difficulty performing maneuvers OR dystonia of the extremity occurring less than 51-75% of the time during waking hours in the past week

4 = ***Severe*** OR ***All the time***: Fixed posturing and unable to perform maneuvers OR dystonia of the extremity occurring more than 75% of the time during waking hours in the past week

7A. RIGHT LOWER EXTREMITY

**Specific Instructions**: Ask the patient (or caregiver) how often, over the past week, has s/he noticed involuntary posturing of the right lower extremity. Ideally, without footwear, observe the patient’s lower extremities with legs dangling then with feet flat on the floor. Ask the patient to walk for at least 5 meters. Observe for any deviations.

0 = ***Normal*** AND ***None***: Absent signs and symptoms

1 = ***Slight*** AND ***Seldom***: Slight posturing and seen only when walking AND dystonia of the extremity occurring less than 25% of the time during waking hours in the past week

2 = ***Mild*** OR ***Often***: Mild posturing and seen even without walking OR dystonia of the extremity occurring 26-50% of the time during waking hours in the past week

3 = ***Moderate*** OR ***Frequent***: Significant posturing is present with difficulty walking OR dystonia of the extremity occurring less than 51-75% of the time during waking hours in the past week

4 = ***Severe*** OR ***All the time***: Fixed posturing and able to ambulate only with assistance OR dystonia of the extremity occurring more than 75% of the time during waking hours in the past week

7B. LEFT LOWER EXTREMITY

**Specific Instructions**: Ask the patient (or caregiver) how often, over the past week, s/he has noticed involuntary posturing of the left lower extremity. Ideally, without footwear, observe the patient’s lower extremities with legs dangling then with feet flat on the floor. Ask the patient to walk for at least 5 meters. Observe for any deviations.

0 = ***Normal*** AND ***None***: Absent signs and symptoms

1 = ***Slight*** AND ***Seldom***: Slight posturing and seen only when walking AND dystonia of the extremity occurring less than 25% of the time during waking hours in the past week

2 = ***Mild*** OR ***Often***: Mild posturing and seen even without walking OR dystonia of the extremity occurring 26-50% of the time during waking hours in the past week

3 = ***Moderate*** OR ***Frequent***: Significant posturing is present with difficulty walking OR dystonia of the extremity occurring less than 51-75% of the time during waking hours in the past week

4 = ***Severe*** OR ***All the time***: Fixed posturing and able to ambulate only with assistance OR dystonia of the extremity occurring more than 75% of the time during waking hours in the past week

8. POSTURE AND BALANCE

**Specific Instruction:** Ask the patient to stand erect then ask the patient to stand on one foot for five seconds. Repeat with the other foot. Score the worse side.

0 = ***Normal***: Able to stand erect and stand on one foot

1 = ***Slight***: Able to stand erect with slight difficulty standing on one foot but still able to

do so for 5 seconds

2 = ***Mild***: Able to stand erect but able to stand on one foot for less than 5 seconds only

3 = ***Moderate***: Some difficulty standing erect or unable to stand on one foot

4 = ***Severe***: Unable to stand on both feet

9. GAIT AND AMBULATION

**Specific Instructions**: Ask the patient to walk for five meters. Observe how the dystonia affects the gait

0 = ***Normal***: Normal gait

1 = ***Slight***: Gait is affected but can walk independently

2 = ***Mild***: Ambulates with difficulty and may require some assistance

3 = ***Moderate***: Can ambulate only with assistance

4 = ***Severe***: Can no longer ambulate even with assistance

**Total Subscale Score: _______**

**PART II: PARKINSONISM**

There are 8 items to be scored. For some items, different body parts or the left and right side may need to be scored separately. Rate the maximal severity of parkinsonism at the time of examination. Specifically for tremors, if maximal severity appears in a body part after you have scored that item, you may go back to re-score.

1. SPEECH

**Specific Instructions:** Ask the patient what s/he had for breakfast today or dinner last night. Ask the patient to recite the months of the year in his/her normal speaking voice.

0 = ***Normal*:** Absent signs and symptoms

1 = ***Slight*:** Slight decrease in volume or slightly slower speech

2 = ***Mild*:** Clear monotony in the voice but speech is not slurred

3 = ***Moderate*:** Slurred speech but still generally understandable

4 = ***Severe*:** Unintelligible speech

1. RESTING TREMORS

**Specific Instructions:** Instruct the patient to put his/ her feet flat on the floor, place his/ her hands on the lap and recite the months of the year starting from December going backwards. Do this on both upper and lower extremities. Be careful in classifying tremors. Parkinsonian tremors are usually rhythmic to-and-fro movements of a body part, typically worse in the resting position. Rate the maximal resting tremor observed. Dystonic tremors, however, are typically irregular in rhythm and vary in amplitude, usually in the setting of an abnormal posturing of the shaking body part. Do not rate these.

A. Face, Lips and Chin:

0 = ***Normal*:** Absent signs and symptoms

1 = ***Slight*:** Tremors are of very low amplitude and present only with reinforcement

2 = ***Mild*:** Tremors are of mild amplitude and persistent OR moderate amplitude but intermittent

3 = ***Moderate*:** Tremors are of moderate amplitude and present most of the time

4 = ***Severe*:** Tremors are of marked amplitude and present most or all the time

B. Upper Extremities:

0 = ***Normal*:** Absent signs and symptoms

1 = ***Slight*:** Tremors are of very low amplitude and present only with reinforcement

2 = ***Mild*:** Tremors are of mild amplitude and persistent OR moderate amplitude but intermittent

3 = ***Moderate*:** Tremors are of moderate amplitude and present most of the time

4 = ***Severe*:** Tremors are of marked amplitude and present most or all the time

C. Lower Extremities

0 = ***Normal*:** Absent signs and symptoms

1 = ***Slight*:** Tremors are of very low amplitude and present only with reinforcement

2 = ***Mild*:** Tremors are of mild amplitude and persistent OR moderate amplitude but intermittent

3 = ***Moderate*:** Tremors are of moderate amplitude and present most of the time

4 = ***Severe*:** Tremors are of marked amplitude and present most or all the time

1. RIGIDITY

**Specific Instructions:** Ask the patient to relax as much as possible. Slowly test the range of motion and tone of each body part. Start with the extremities and end with the neck region. With each maneuver, if the tone is normal and with full range of motion, ask the patient to open and close the contralateral hand. Do this on both upper and lower extremities and the neck. Rate the maximum rigidity observed within the body region.

A. Upper Extremities

0 = ***Normal*:** Absent signs and symptoms

1 = ***Slight*:** Increased tone detected only with activation procedures

2 = ***Mild*:** Mild rigidity appreciated even without activation procedures

3 = ***Moderate*:** Moderate rigidity, full range of motion achieved with some difficulty

4 = ***Severe*:** Full range of motion can barely be achieved

B. Lower Extremities

0 = ***Normal*:** Absent signs and symptoms

1 = ***Slight*:** Increased tone detected only with activation procedures

2 = ***Mild*:** Mild rigidity appreciated even without activation procedures

3 = ***Moderate*:** Moderate rigidity, full range of motion achieved with some difficulty

4 = ***Severe*:** Full range of motion can barely be achieved

C. Neck

0 = ***Normal*:** Absent signs and symptoms

1 = ***Slight*:** Increased tone detected only with activation procedures

2 = ***Mild*:** Mild rigidity appreciated even without activation procedures

3 = ***Moderate*:** Moderate rigidity, full range of motion achieved with some difficulty

4 = ***Severe*:** Full range of motion can barely be achieved

1. FINGER TAPS

**Specific Instructions: A**sk the patient to tap thumb with the index finger in rapid succession 10 times as fast and as wide as possible; demonstrate then ask the patient to do the maneuver on his/ her own.

A. Right

0 = ***Normal*:** Absent signs and symptoms

1 = ***Slight*:** Slight slowing and/ or reduction in amplitude is noted

2 = ***Mild*:** Mild but clear slowing observed and/or occasional arrests in movements

3 = ***Moderate*:** Moderate slowness with frequent hesitation/ arrests

4 = ***Severe*:** Can barely perform the task

B. Left

0 = ***Normal*:** Absent signs and symptoms

1 = ***Slight*:** Slight slowing and/ or reduction in amplitude is noted

2 = ***Mild*:** Mild but clear slowing observed and/or occasional arrests in movements

3 = ***Moderate*:** Moderate slowness with frequent hesitation/ arrests

4 = ***Severe*:** Can barely perform the task

1. ALTERNATING HAND MOVEMENTS

**Specific Instructions:** Ask the patient to do alternating pronation and supination motions of the hands in rapid succession 10 times, one hand at a time, as full and as fast as possible. Demonstrate then ask the patient to do the maneuver on his/ her own.

A. Right

0 = ***Normal*:** Absent signs and symptoms

1 = ***Slight*:** Slight slowing and/ or reduction in amplitude is noted

2 = ***Mild*:** Mild but clear slowing observed and/or occasional arrests in movements

3 = ***Moderate*:** Moderate slowness with frequent hesitation/ arrests

4 = ***Severe*:** Can barely perform the task

B. Left

0 = ***Normal*:** Absent signs and symptoms

1 = ***Slight*:** Slight slowing and/ or reduction in amplitude is noted

2 = ***Mild*:** Mild but clear slowing observed and/or occasional arrests in movements

3 = ***Moderate*:** Moderate slowness with frequent hesitation/ arrests

4 = ***Severe*:** Can barely perform the task

1. LEG AGILITY

**Specific Instructions:** Ask the patient to tap his heel on the ground in rapid succession 10 times as high and as fast as possible, lifting up the entire leg. Do this one side at a time; demonstrate it, then ask the patient to do this on his/ her own.

A. Right

0 = ***Normal*:** Absent signs and symptoms

1 = ***Slight*:** Slight slowing and/ or reduction in amplitude is noted

2 = ***Mild*:** Mild but clear slowing observed and/or occasional arrests in movements

3 = ***Moderate*:** Moderate slowness with frequent hesitation/ arrests

4 = ***Severe*:** Can barely perform the task

B. Left

0 = ***Normal*:** Absent signs and symptoms

1 = ***Slight*:** Slight slowing and/ or reduction in amplitude is noted

2 = ***Mild*:** Mild but clear slowing observed and/or occasional arrests in movements

3 = ***Moderate*:** Moderate slowness with frequent hesitation/ arrests

4 = ***Severe*:** Can barely perform the task

1. ARISING FROM THE CHAIR

**Specific Instructions:** While seated, ask patient to fold his arms across his/ her chest and attempt to quickly stand up.

0 = ***Normal*:** Absent signs and symptoms

1 = ***Slight*:** Slow in standing, or may need more than 1 attempt but can do so without using the arm rest of the chair

2 = ***Mild*:** Needs to push self from the arm rest of the chair

3 = ***Moderate*:** Needs more than 1 attempt despite using the arm rest of the chair but still able to do without help

4 = ***Severe*:** Unable to arise without help

1. GAIT FREEZING AND SHUFFLING

**Specific Instructions:** Ask the patient to walk 5 meters and turn a couple of times. Observe for shuffling, freezing and hesitation.)

0 = ***Normal*:** No shuffling, freezing or hesitation

1 = ***Slight*:** Rare difficulty in walking, shuffling or hesitation is noted, but generally steady and confident gait

2 = ***Mild*:** Mild difficulty in walking, some short festination or hesitation, or turns *en bloc* but still able to walk without assistance

3 = ***Moderate*:** Moderate difficulty in walking requiring a walking aid (eg. cane, walker, etc.) but still able to do so independently

4 = ***Severe*:** Wheel chair- or bed-bound unless aided

**Total Subscale Score: _______**

**PART III: NON-MOTOR FEATURES**

This subscale is divided into two parts. Part IIIA (BEHAVIORAL segment) is Clinician-administered, and Part IIIB (NON-BEHAVIORAL segment) is Patient- or Caregiver-administered.

**Part IIIA (Behavioral Segment, Clinician-Administered)**

There are 5 items to be scored

**Primary source of information *(circle one)*:**

PATIENT CAREGIVER BOTH (IN EQUAL PORTION)

1. COGNITION

**Instruction to Examiner:** Consider all types of altered level of cognitive function, including cognitive slowing, impaired reasoning, memory loss, deficits in attention and orientation. Then, rate their duration and interference with patient’s ability to carry out daily routines and engage in social interactions.

**To be read to patients (and caregivers):** *“Over the past week, have you had problems remembering things, following conversations, paying attention, thinking clearly, or finding your way around the house or in town? How much do these problems with memory and concentration bother you or affect your day-to-day activities?”* [If yes, examiner asks patient or caregiver to elaborate and probes for information]

0 = ***Normal*:** No signs/symptoms of cognitive impairment for the past week

1 = ***Slight*:** Cognitive impairment recognized by patient and/or caregiver but creates

no interference in the patient’s ability to carry out daily routines and

engage in social interactions

2 = ***Mild*:** Cognitive impairment occasionally interferes but does not prevent the

patient’s ability to carry out daily routines and engage in social interactions

3 = ***Moderate*:** Cognitive impairment significantly interferes, but does not totally prevent the patient’s ability to carry out daily routines and engage in social interactions

4 = ***Severe*:** Cognitive impairment totally prevents the patient’s ability to carry out

daily routines and engage in social interactions

1. APATHY

**Instruction to Examiner:** Consider level of spontaneous activity, assertiveness, motivation and initiative, and rate its and interference with patient’s ability to carry out daily routines and engage in social interactions. In this category, the examiner should attempt to distinguish between apathy and similar symptoms that are best explained by depression.

**To be read to patients (and caregivers):** *“Over the past week, did you have difficulty starting/initiating or have you lost interest in doing your usual or recreational activities? Have you lost interest in interacting and socializing with people? How much has your difficulty initiating or lack of interest affected your day-to-day activities and social interactions?”* [If yes, Examiner asks patient or caregiver to elaborate and probes for information]

0 = ***Normal*:** No signs/ symptoms of apathy over the past week

1 = ***Slight*:** Apathy recognized by patient and/or caregiver but creates no interference in the patient’s ability to carry out daily routines and engage in social

interactions

2 = ***Mild*:** Apathy occasionally interferes but does not prevent the patient’s ability to carry out daily routines and engage in social interactions

3 = ***Moderate*:** Apathy significantly interferes, but does not totally prevent the patient’s

ability to carry out daily routines and engage in social interactions

4 = ***Severe*:** Apathy prevents the patient’s ability to carry out daily routines and engage

in social interactions

1. ANXIETY

**Instruction to Examiner:** Determine nervous, tense, worried or anxious feelings (including panic attacks) over the past week and rate their duration and interference with patient’s ability to carry out daily routines and engage in social interactions.

**To be read to the patient (and caregiver):** *“Over the past week, have you felt nervous, anxious, or uneasy? Did you experience palpitations, shortness of breath, and other physical symptoms in association with your nervousness? How has this affected your day-to-day activities and your social interactions?”*

0 = ***Normal:*** no signs/symptoms of anxious feelings over the past week

1 = ***Slight*:** Slight or intermittent anxiety feelings that does not cause interference in the patient’s ability to carry out daily routines and engage in social interactions

2 = ***Mild*:** Clear anxiety feelings that occasionally interfere but do not prevent the patient’s ability to carry out daily routines and engage in social

interactions

3 = ***Moderate*:** Significant anxiety that frequently interferes, but do not totally prevent

the patient’s ability to carry out daily routines and engage in social

interactions

4 = ***Severe*:** Severe and persistent anxiety feelings that prevent the patient’s ability to

carry out daily routines and engage in social interactions

1. DEPRESSION

**Instruction to Examiner:** Consider low mood, sadness, hopelessness, guilt, feelings of emptiness, or loss of enjoyment. If the patient is SUICIDAL (whether planned, attempted or failed), Category 4 [severe] should be automatically chosen.

**To be read to the patient (and caregiver):** “*Over the past week, did you feel sad, ‘blue’, or hopeless? Did you feel guilty sometimes that you have not lived up to expectations or you are causing undue burden to others? Did you sometimes wish that life as not worth living? Did you ever act on these thoughts?”*

0 = ***Normal*:** No signs/symptoms of depressed mood over the past week)

1 = ***Slight*:** Slightly and transiently depressed mood does not cause interference in the

patient’s ability to carry out daily routines and engage in social

interactions

2 = ***Mild*:** Mildly depressed mood occasionally interferes but does not prevent the

patient’s ability to carry out daily routines and engage in social

interactions

3 = ***Moderate*:** Significantly depressed mood most of the time that frequently

interferes, but does not totally prevent the patient’s ability to carry out daily

routines and engage in social interactions

4 = ***Severe*:** Severe and persistent depressed mood that prevents the patient’s ability to

carry out daily routines and engage in social interactions

1. IRRITABILITY AND AGGRESSION

**Instruction to Examiner:** Consider the patient’s dealing with caregiver and other people around. This includes lack of impulse control, unpleasant, irritable, and aggressive behaviors. Then rate their duration and interference with patient’s ability to carry out daily routines and engage in social interactions.

**To be read to the patient (and caregiver):** *“Over the past week, have you felt angry or irritable? Did you feel you were ‘short fused’ and easily upset and irritated over minor things? Did you constantly argue or yell or hit people around you? Did you have difficulty controlling your emotions or desires?*

0: ***Normal*:** No signs/symptoms of unpleasant behavior for the past week

1 = ***Slight***: Slight and occasional unpleasant behavior but does not cause interference in

the patient’s ability to engage in social interactions

2 = ***Mild*:** Mild unpleasant behavior that has somewhat affects the patient’s ability to

engage in social interactions

3 = ***Moderate*:** Unpleasant behavior that significantly affects the patient’s ability to

engage in social interactions

4 = ***Severe*:** Severe and persistent unpleasant behavior with physical aggressiveness

and/ or verbal abusiveness that prevents any meaningful social interaction

**Total subscale score: _______**

**Part IIIB (Non-Behavioral Segment; Patient/Caregiver-Administered)**

There are 5 items to be scored.

**Patient (or Caregiver) Instructions:** *Sometimes, people with your condition, experience certain symptoms such as pain, fatigue, and problems with sleep and urination. We would like to ask you questions about these symptoms. Please choose the best response that describes how you have felt ON AVERAGE during the PAST WEEK including today. It is possible that some of these questions will not apply to you now or ever, so please do not worry as you respond to these questions. If you are not experiencing these problems, you can simply respond NO to the questions.*

**Who answered this form today? *(circle one)*:**

PATIENT CAREGIVER

If caregiver: What is your relationship to the patient? _____________________

1. SLEEP DISTURBACE

*Over the past week, how would you rate your ability to fall asleep at night, and your ability to stay asleep? Did you wake up in the morning feeling rested? Did you have to take sleeping pills to help you sleep or stay asleep?*

0 = I have no sleep problems in the past week and I feel rested when I wake up.

1 = I had a slight problem sleeping in 1 or 2 nights only in the past week.

2 = I had difficulty sleeping about half the nights in the past week.

3 = I had difficulty sleeping most of the nights in the past week and I don’t feel rested

when I woke up. Sometimes, I have to take sleeping pills to fall asleep.

4 = I am unable to sleep without my sleeping pills.

1. PAIN AND OTHER SENSATIONS

*Over the past week, have you had uncomfortable feelings in your body such as pain, aches, tingling or cramps? How bothersome were they? Did it prevent you from doing your day-to-day activities or interfere with your sleep?*

0 = I had no uncomfortable feelings/ pain in the past week.

1 = I experienced mild and transient uncomfortable feelings/ pain in the past week but it

did not interfere with me sleep or day-to-day activities.

2 = I experienced occasional uncomfortable feelings/pain that somewhat interfered with my sleep or day-to-day activities.

3 = I experienced uncomfortable feelings/ pain on most days in the past week and it significantly interfered with my sleep or my day-to-day activities.

4 = I was hardly able to sleep or do any day-to-day activities in the past week because of the uncomfortable feelings/ pain that I felt.

1. BLADDER INCONTINENCE

*Over the past week, have you had trouble with urine control? How often did you feel the urgent need to urinate? Did you feel the need to urinate very often? Did you have some urine accidents?*

0 = I had no urine control problems in the past week.

1 = Once or twice only in the past week, I felt the urgent need to urinate or I woke up several times during the night to urinate, but I did not have any urine accidents.

2 = On at least half the days or nights in the past week, I had urinary problems, but I did not have any urinary accidents.

3 = I experienced urinary problems almost every day in the past week, and occasionally, I would have an accident.

4 = I cannot control my urine and I use a protective garment or have a bladder tube.

1. FATIGUE

*Over the past week, on average, did you feel tired or worn out? Did this affect your day-to- day activities?*

0 = I did not experience excessive tiredness in the past week.

1 = I experienced slight fatigue in the past week. However, it does not create interference in my ability to carry out daily routines.

2 = The fatigue I experienced in the past week occasionally interfered but did not

prevent my ability to carry out daily routines.

3 = The fatigue I experienced in the past week caused me a lot of problems and significantly interfered with my ability to carry out daily routines.

4 = I could hardly do any of my activities in the past week because of fatigue.

1. SALIVA & DROOLING

*Over the past week, did you notice some drooling?*

0 = I had no problems with excess saliva and drooling.

1 = I could feel excess saliva in the mouth but did not drool during sleep or when awake.

2 = I had some drooling during sleep, but none when I was awake.

3 = I had some drooling even when I was awake.

4 = I was constantly drooling.

**Total subscale score: _______**

**PART IV: ACTIVITIES OF DAILY LIVING (Patient/Caregiver-Administered)**

There are 10 items to be scored.

**Patient (or Caregiver) Instructions:** *We would like to ask you questions about how certain problems may be affecting your day-to-day activities. Please choose the best response that describes how you felt ON AVERAGE during the PAST WEEK including today. It is possible that some of these questions will not apply to you now or ever, so please do not worry as you respond to these questions. If you are not experiencing these problems you can simply respond NO to the questions.*

**Who answered this form today? *(circle one)*:**

PATIENT CAREGIVER

If caregiver: What is your relationship to the patient? __________________________

1. SPEECH

*Over the past week, did you have problems with your speech?*

0 = Not at all (no problems).

1 = My speech was soft, slurred or uneven, but I was not asked to repeat myself.

2 = My speech caused people to asked me to occasionally repeat myself.

3 = My speech was unclear enough that others asked me to repeat myself almost every day.

4 = My speech could not be understood almost all the time.

1. CHEWING AND SWALLOWING

*Over the past week, have you had problems drinking, swallowing or eating? Do you need pills cut or crushed or your food made to be soft, chopped or blended?*

0 =No problems

1 = I was aware of slowness when chewing or increased effort when swallowing but I did

not choke and I did not need to have food specially prepared or modified.

2 = I choked once or twice in the past week but my food or drink has not been modified.

3 = I choke occasionally at least once a week and my food had to be modified to prevent choking.

4 = Because of drinking, chewing or swallowing problems, I needed a feeding tube.

1. EATING/FEEDING

*Over the past week, have you usually had trouble handling your food and using eating utensils? Do you have trouble handling finger foods or using forks, knives, spoons or chopsticks?*

0 = Not at all (no problems).

1 = I was somewhat slow, but I do not need any help handling my food and have not had

food spills while eating.

2 = I was slow with my eating and had occasional food spills. I may have needed help with a few tasks such as cutting food into smaller portions.

3 = I needed help with many eating tasks but could manage some alone.

4 = I was unable to use my hands and was only able to get food using my mouth or I needed

help for most or all eating tasks.

1. DRESSING

*Over the past week, did you have problems dressing? Were you slow or did you need help with buttoning, using zippers, putting on or taking off?*

0 = Not at all (no problems).

1 = I was somewhat slow but I did not need help.

2 = I was slow and needed help for a few dressing tasks (buttons).

3 = I needed help for many dressing tasks.

4 = I needed help for most or all dressing tasks.

1. HYGIENE

*Over the past week, did you need help with washing, bathing, shaving, brushing teeth, combing your hair or with other personal hygiene?*

0 = Not at all (no problems).

1 = I was somewhat slow but I did not need any help.

3 = I needed someone else to help me with some hygiene tasks.

4 = I needed help for many hygiene tasks.

5 = I needed help for most or all of my hygiene tasks.

1. HANDWRITING

*Over the past week, did people have trouble reading your handwriting?*

0 = Not at all (no problems).

1 = My writing was slow, clumsy or uneven, but all words were easy to read.

2 = Some words were difficult to read.

3 = Many words were difficult to read.

4 = Most or all words could not be read.

1. DOING HOBBIES AND OTHER ACTIVITIES

*Over the past week, did you have trouble doing your hobbies or other things that you like to do?*

0 = Not at all (no problems).

1 = I was a bit slow but did these activities easily.

2 = I had some difficulty doing these activities.

3 = I had major problems doing these activities, but still did most.

4 = I was unable to do most or all of these activities.

1. TURNING IN BED

*Over the past week, did you have trouble turning over in bed?*

0 = Not at all (no problems).

1 = I had a bit of trouble turning, but I did not need any help.

2 = I had a lot of trouble turning and needed occasional help from someone else.

3 = To turn over I often needed help from someone else.

4 = I was unable to turn over without help from someone else.

1. TRANSFERING/GETTING OUT OF BED, A DEEP CHAIR OR VEHICLE

*Over the past week, did you have trouble getting out of bed, a vehicle, or a deep chair?*

0 = Not at all (no problems).

1 = I was slow or awkward, but I usually could do it on my first try.

2 = I needed more than one try to get up or needed occasional help.

3 = I sometimes needed help to get up, but most of the times I could still do it on my own.

4 =I needed help most or all of the time.

1. WALKING AND BALANCE

*Over the past week, did you have problems with balance and walking?*

0 = Not at all (no problems).

1 = I was slightly slow or may have dragged a leg. I never used a walking aid (cane,

walker).

2 = I occasionally used a walking aid, but I did not need any help from another person.

3 = I usually used a walking aid to walk safely without falling. However, I did not usually

need the support of another person.

4 = I usually used the support of another person to walk safely without falling.

**Total Subscale Score: ________**

**TOTAL SCORE:** _______ + _______ + _______ + _______ + _______ = _______

I II IIIA IIIB IV Total

**V Global Rating**

**Global Severity**: Interviewer's judgment of the overall severity of the patient's illness. This should focus on the overall burden of the patient’s symptoms with respect to its impact on daily functioning (e.g., sleep; ability to work; ability to relate to others) and/or the overall severity of illness/symptoms. In the instance that severity is greater than interference (or interference greater than severity), rate according to the most severe or impaired domain. Rated from 1 (no illness) to 7 (most severe patient seen). (Consider the degree of distress reported by the patient or caregiver, the symptoms observed, and the functional impairment reported. Your judgment is required both in averaging this data as well as weighing the reliability or accuracy of the data obtained. This judgment is based on information obtained during the interview.)

- - 1. NORMAL, NOT ILL: no illness
    2. BORDERLINE: Subtle illness with minimal, if any functional impairment
    3. MILDLY ILL: Clearly established symptoms with minimal, if any difficulty in function
    4. MODERATELY ILL: Overt symptoms causing noticeable but modest functional impairment and difficulty with goal directed activities
    5. MARKEDLY ILL: Intrusive symptomatology that distinctly impairs social/occupational function and goal directed activities
    6. SEVERELY ILL: Disruptive symptoms; function is frequently influenced by symptomatology and often requires supervision
    7. EXTREMELY ILL: Symptoms drastically interferes in many life functions; incapable of goal-directed activities

**Global Improvement Symptoms**: Rate total overall improvement present *since the initial rating* whether or not, in your judgment, it is due to treatment.

- - 1. VERY MUCH IMPROVED: Nearly all symptoms got better; good level of functioning; minimal symptoms; represents a very substantial change
    2. MUCH IMPROVED: Notably better with significant reduction of symptoms; increase in the level of functioning but some symptoms remain
    3. MINIMALLY IMPROVED: Slightly better with little or no clinically meaningful reduction of symptoms; represents very little change in basic clinical status, level of care, or functional capacity
    4. NO CHANGE: Symptoms remain essentially unchanged
    5. MINIMALLY WORSE: Slightly worse but may not be clinically meaningful; may represent very little change in basic clinical status or functional capacity
    6. MUCH WORSE: Clinically significant increase in symptoms and diminished functioning
    7. VERY MUCH WORSE: Severe exacerbation of symptoms and loss of functioning
